# Supplementary material for: Complex‐centric proteome profiling by SEC‐SWATH‐MS
Source: Mol Syst Biol. 2019 Jan 14;15(1):e8438. doi: 10.15252/msb.20188438 (PMC6346213; doi:10.15252/msb.20188438)
Supplement: Supplementary file 8 — Dataset EV7 [file MSB-15-e8438-s008.zip › feature_plots_string/P61619.pdf]

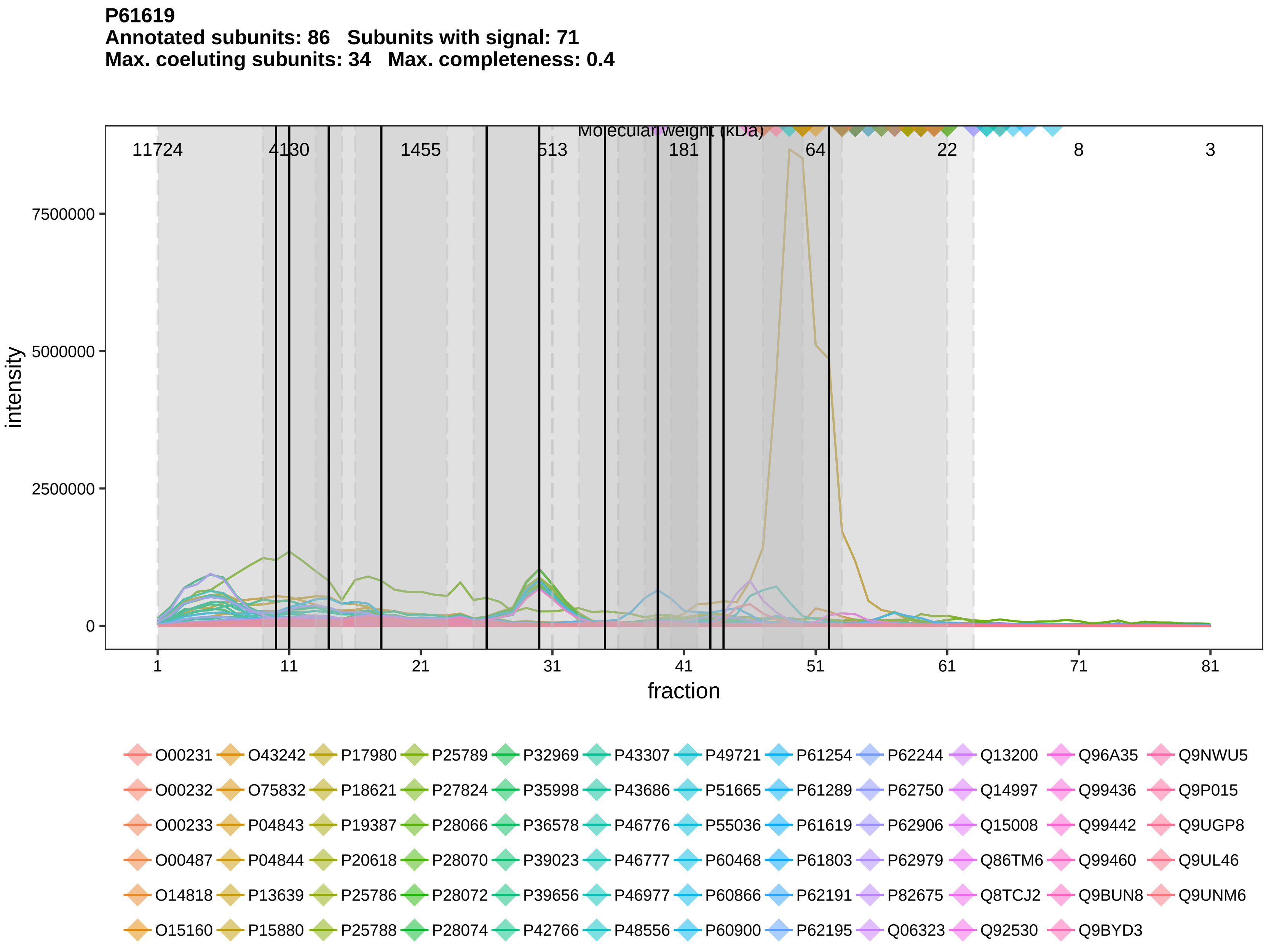
P61619
Annotated subunits: 86 Subunits with signal: 71
Max. coeluting subunits: 34 Max. completeness: 0.4
Molecular weight (kDa)
11724
4130
1455
513
181
64
22
8
3
7500000
5000000
2500000
0
intensity
1
11
21
31
41
51
61
71
81
fraction
O00231 O43242 P17980 P25789 P32969 P43307 P49721 P61254 P62244 Q13200 Q96A35 Q9NWU5
O00232 O75832 P18621 P27824 P35998 P43686 P51665 P61289 P62750 Q14997 Q99436 Q9P015
O00233 P04843 P19387 P28066 P36578 P46776 P55036 P61619 P62906 Q15008 Q99442 Q9UGP8
O00487 P04844 P20618 P28070 P39023 P46777 P60468 P61803 P62979 Q86TM6 Q99460 Q9UL46
O14818 P13639 P25786 P28072 P39656 P46977 P60866 P62191 P82675 Q8TCJ2 Q9BUN8 Q9UNM6
O15160 P15880 P25788 P28074 P42766 P48556 P60900 P62195 Q06323 Q92530 Q9BYD3
